# The “Surprise Question” in Neurorehabilitation—Prognosis Estimation by Neurologist and Palliative Care Physician; a Longitudinal, Prospective, Observational Study

**DOI:** 10.3389/fneur.2018.00792

**Published:** 2018-09-24

**Authors:** Markus Ebke, Andreas Koch, Kim Dillen, Ingrid Becker, Raymond Voltz, Heidrun Golla

**Affiliations:** ^1^Neurological Centre for Rehabilitation-MEDIAN-Clinics, Bad Salzuflen, Germany; ^2^Dr. Becker Rhein Sieg Clinic, Nümbrecht, Germany; ^3^Department of Palliative Medicine, University Hospital of Cologne, Cologne, Germany; ^4^Institute of Medical Statistics and Computational Biology (IMSB), University of Cologne, Cologne, Germany; ^5^Center for Integrated Oncology Cologne/Bonn, Cologne, Germany; ^6^Center for Clinical Trials, University of Cologne, Cologne, Germany; ^7^Medical Faculty, Center for Health Services Research (ZVFK), University of Cologne, Cologne, Germany

**Keywords:** surprise question, neurorehabilitation, palliative care, observational study, prognosis, outcome measurement

## Abstract

**Background:** The 12-months “surprise” question (12-SQ) for estimating prognosis and the need for integrating palliative care (PC) services has not yet been investigated for neurological patients.

**Objective:** Test the value of the 12-SQ on a sample of neurorehabilitation patients.

**Methods:** All patients newly registered in the Department of Neurorehabilitation, Dr. Becker Rhein-Sieg-Clinic (8/2016-03/2017) were asked to participate. The treating neurorehabilitation physicians (NP) and an external consulting PC physician (PCP) independently estimated patients' prognosis using the 12-SQ; while symptom burden was independently assessed using the standardized palliative outcome measurement HOPE-SP-CL, a set of additional neurological issues, and ECOG. Follow-up with consenting patients 12 months later was via telephone. Descriptive and inferential statistics were utilized in data analysis.

**Results:** Of 634 patients, 279 (44%) patients (male: 57.7%, female: 42.3%; mean age: 63 ± 14) (or, alternatively, their legal representative) consented and were assessed at baseline. Per patient NP and PCP both answered the 12-SQ with “Yes” (164), with “No” (42), or had different opinions (73). The “No” group displayed the highest symptom burden on all three measures for both disciplines. Overall, PCP scored higher (i.e., worse) than NP on all measures used. Follow-up was possible for 236 (drop-out: 15.4%) patients (deceased: 34 (14.4%), alive: 202 (85.6%)). Baseline scores on all measures were higher for deceased patients compared to those still living. Prognostic characteristics were: *sensitivity:* NP 50%, PCP 67.6%; *specificity:* NP 86.1%, PCP 70.3%, *p* < 0.001; *positive predictive value:* NP 37.8%, PCP 27.7%; *negative predictive value:* NP 91.1%, PCP 92.8%; *area under the curve:* NP 0.68, PCP 0.69; *success rate:* NP 80.9%, PCP 69.9%, *p* = 0.002. Regression analysis indicated that age, dysphagia and overburdening of family (NP answering the 12-SQ), dysphagia and rehabilitation phase (PCP answering the 12-SQ) were associated with increased likelihood of dying within 12 months. Without the 12-SQ as relevant predictor, age, dysphagia and ECOG were significant predictors (NP and PCP).

**Conclusion:** Combining the 12-SQ with a measurement assessing PC and neurological issues could potentially improve the 12-SQ's predictive performance of 12-month survival and help to identify when to initiate the PC approach. Clinical experiences influence assessment and prognosis estimation.

## Introduction

For predicting the point at which to introduce palliative care for incurable cancer patients the German national S3 palliative guideline^1^ recommends using the 12-months “surprise” question (12-SQ) (Would you be surprised if your patient would die within the next 12 months?). A “No” response (i.e., a poor prognosis) indicates that the assessor considers it a possibility that the patient could die within the next 12 months and, thus, palliative care should be initiated promptly.

The various disease entities to which the SQ has been applied thus far include cancer (1–5), chronic obstructive pulmonary disease (COPD) (6), nephrological diseases (7–13), as well as pediatric palliative care (14), intensive care (15), emergency care (16, 17), and in elderly care (17–19). The SQ has yet to be applied to neurological care where patients are characterized by different disease trajectories compared to other disease entities, especially cancer patients. Prognosis estimation is therefore challenging (20) and a suitable prognostic instrument would aid in estimating lifespan and indicating when best to initiate the palliative care approach for these patients.

A recent systematic review and meta-analysis revealed that the SQ it is not an ideal diagnostic tool for predicting one-year mortality, especially among non-cancer patients (21). Rather, the use of additional parameters seems warranted (22, 23).

Thus, in addition to the 12-SQ, supplementary assessment tools focusing on patients' symptom burden were employed for this study. Typical palliative care assessment tools, including the German Hospice and Palliative Care Evaluation initiative (HOPE = HOspiz- und PalliativErhebung) (24) and the internationally used palliative outcome scale (POS) (25, 26) were developed for patients suffering from later stage cancer diseases. This is not surprising, as presently, palliative and hospice care structures primarily care for advanced cancer patients^2^ (27, 28), although the portion of cancer patients has slightly decreased (95% in 2005 vs. 76% in 2017 (see^2^) with respect to other disease entities such as neurological conditions, COPD, nephrological diseases or chronic heart failure (see^2^). For example, patients cared for in German palliative and hospice care structures suffering from nervous system diseases represented 4.8% in 2017 compared to only 0.8% in 2005 (see^2^). Despite this slight increase in the number of neurological patients in German palliative and hospice care, the current, rather small, percentage is still astonishing considering the great number and variety of neurological diseases, among them long-term neurological conditions (LTNC) which present with a high symptom burden. Given these cases are mostly incurable, symptom relief, and enhanced quality of life are the leading therapeutic goals in treating these patients, according to the World Health Organization (WHO)^3^. Using a combined neurorehabilitation and palliative care approach, neuropalliative rehabilitation for LTNC is on the way to becoming integrated into care in the UK (29–31), in contrast to Germany. Symptoms and complaints among neurological and cancer patients are in part fairly similar but may differ in their manifestation and certain issues clearly transcend those of cancer patients, presenting distinct challenges for such patients (32–40). Therefore, the neurorehabilitation study population was characterized utilizing a combination of a standard palliative care assessment tool (HOPE including ECOG) and an additional list of items representative for neurological disease entities as revealed from longstanding clinical experience, literature (32–40) and a previous study on glioblastoma (41). In addition to using the 12-SQ, this detailed characterization can help to identify further prognostic criteria of neurological patients, which may lead to improved prognostic accuracy of the 12-SQ, an approach in accordance with other studies commending additional tools other than the 12-SQ to predict mortality (22, 23).

The objective of this study was to investigate prognostic criteria for neurological patients. For the first time, the suitability of the 12-SQ for neurological patients was prospectively investigated, combining it with an assessment merging palliative care and neurological issues.

Patients were recruited from among those newly registered at a neurorehabilitation clinic providing care to a broad range of neurological disease entities. An important secondary goal was to examine whether the professional background—being a neurorehabilitation physician (NP) or being a palliative care physician (PCP) with no neurology background—played a role in assessment and prognosis estimation. This is a critical issue as a consultant palliative care service is not typically integrated in neurorehabilitation clinics and NP and their teams must make decisions on their own. On the other hand, the PCP might also care for neurological patients but only a small percentage of them are trained in neurology. Thus a complementary approach suggests itself, one that includes the professional assessment of both, NP and PCP.

### In summary, aims of the study were

#### Primary objective

Is the 12-SQ suitable for prognosis estimation with neurological patients?

#### Secondary objectives

Does prognosis estimation depend on the physicians' background (NP vs. PCP)?

How is the study population characterized and assessed by both, NP and PCP?

How are the patients who died within this 12 month period actually characterized? Can factors be deduced which would help estimate the prognosis of these patients alone or in combination with the 12-SQ?

## Materials and methods

### Study design

This is a longitudinal, prospective, observational study. The recruitment period encompassed August 10, 2016–March 10, 2017. The follow-up period extended until March 10, 2018; 12 months later.

### Study participants

All newly admitted patients (permitted age range 18–100 years, all genders) then in treatment at the Dr. Becker Rhein-Sieg-Klinik, Department of Neurorehabilitation (phase B, C, D; a German classification system characterizing type and intensity of neurological rehabilitation) during the recruitment period were enrolled in the study after providing their informed written consent (or alternatively via their legal representative). The local ethics committees of the North Rhine Medical Chamber and of the University Hospital of Cologne approved the study (#16–118).

### Data collection

For quality assurance and to enable a patient-oriented care post-hospital discharge at the Dr. Becker Rhein-Sieg-Klinik, department for neurorehabilitation, an estimation of prognosis using the 12-SQ and an assessment of symptom burden was implemented into the clinical routine.

#### “surprise”-question

At time of admission, treating NP - neurologists with additional neurorehabilitation expertise, but no specialist training in palliative care—as well as an external consulting PCP—with no neurological training—responded independently to the 12-SQ. The PCP visited the Department of Neurorehabilitation once a week.

#### Assessment of symptom burden

Concurrently to answering the 12-SQ, both NP and PCP also independently assessed the symptom burden of the neurological patients utilizing the core documentation of the German Hospice and Palliative Care Evaluation initiative (HOPE), the HOPE symptom and problem checklist (HOPE-SP-CL) (24) including the Eastern Cooperative Oncology Group (ECOG) Performance Status scale. The HOPE-SP-CL consists of 17 items and assesses symptoms and problems representative for cancer patients in palliative care (24). Single items are scaled using a 4-point grading scale (0 = none, 1 = mild, 2 = moderate, 3 = severe) (possible total score: 0–51) (24). The ECOG Performance Status scale is a 5-point grading scale ranging from 0 (normal activity) to 4 (care-dependent, totally confined to bed).

A list of symptoms which might be of special importance for neurological patients who have or might develop palliative care needs was added to account for the particularities of the neurological patients' symptom burden (32–40). This “neuro supplement” was derived from clinical experience and existing literature (32–40). Augmenting this was a preliminary study (41) on assessing palliative care issues utilizing standardized outcome measurements (HOPE-SP-CL (24, 42), the POS (palliative outcome scale) (25, 26) as well as an open interview part which included symptoms not covered by these assessment tools. The neuro supplement scale derived from this comprises 13 items. Following the HOPE-SP-CL scale, single items of the neuro supplement are scaled using a 4-point grading Likert scale (0 = none, 1 = mild, 2 = moderate, 3 = severe) (possible total score: 0–39). All 3 scales (HOPE-SP-CL, ECOG, neuro supplement) combined in this study result in a possible total score ranging from 0 to 94.

#### Follow-up

Twelve months after answering the 12-SQ, patients (or alternatively their legal representative) were contacted via telephone by NP (ME) or PCP (AK) to find out whether patients were still alive.

### Statistical analysis

Distribution of age, gender, rehabilitation phase, main, and secondary diagnoses, results from the clinical assessment (12-SQ-answer, HOPE-SP-CL, ECOG, neuro supplement) were analyzed descriptively to characterize the study population at baseline.

The classification of patients was done in two separate steps. First, we used each physician's independent response to the 12-SQ, assigning patients to either the “Yes” group or the “No” group. Next, we combined the physicians' responses and allocated patients into three individual groups. Those who were given a good prognosis by both physicians were classified into the 12-SQ “Yes” group, those given a poor prognosis by both physicians were categorized as the 12-SQ “No” group, and those with contrary ratings were classified as the 12-SQ “Discordant”-group. This classification into three groups allowed us to characterize the study population (statistical details below) when the consensus of both physicians was used (secondary objective).

Prior to all analyses, the Kolmogorov-Smirnov Test was applied to assess normality for all relevant variables.

To investigate whether the 12-SQ can be used as a prognostic indicator for neurological patients (primary objective), the predictive power of the 12-SQ was determined using receiver operating characteristics (ROC) curves to assess sensitivity (proportion of patients who died within 12 months and were given a poor prognosis), specificity (proportion of patients who survived over 12 months and were given a good prognosis), positive predictive value (PPV, proportion of poor prognoses correctly predicting death within 12 months), negative predictive value (NPV, proportion of good prognoses correctly predicting survival over 12 months), and the area under the curve (AUC, function of both sensitivity and specificity measuring the predictive accuracy). Similarly, we examined the success rates of both physicians; defined as percentage of correct predictions accounting for all possible outcomes. The differences in prognostic accuracy proportions between the NP and PCP were assessed with the McNemar χ^2^ test as appropriate (secondary objective) (43).

Group differences of demographic and clinical data were tested with the Mann-Whitney-U test and the Kruskall-Wallis test (for two and three groups, respectively) for continuous measures and a χ^2^ test for dichotomous measures. *Post-hoc* tests were corrected for multiple comparisons using the false discovery rate (FDR) at *p* < 0.05 (44).

To determine independent predictors of 12-month mortality (secondary objective), binary logistic regression analyses were performed for both NP and PCP. In addition to the 12-SQ, age, gender, main and secondary diagnoses, frequency of secondary diagnoses, rehabilitation phase, HOPE-SP-CL single items, neuro supplement single items and the ECOG score were included in the model. A univariate regression was constructed first. Resulting predictors with a *p*-value of < 0.1 were then included in the multivariable regression. To determine which variables best predicted 12-month mortality in the presence and absence of the 12-SQ, we selected statistically significant predictors of the multivariable regression (at *p* < 0.05) for the final model and compared their prognostic accuracy indices to those of the 12-SQ as a stand-alone predictor.

Statistical analysis was performed using the Statistical Package for the Social Sciences (SPSS) software (v. 25, Inc, Chicago, IL).

## Results

### Study participation and follow-up

**Figure 1:**
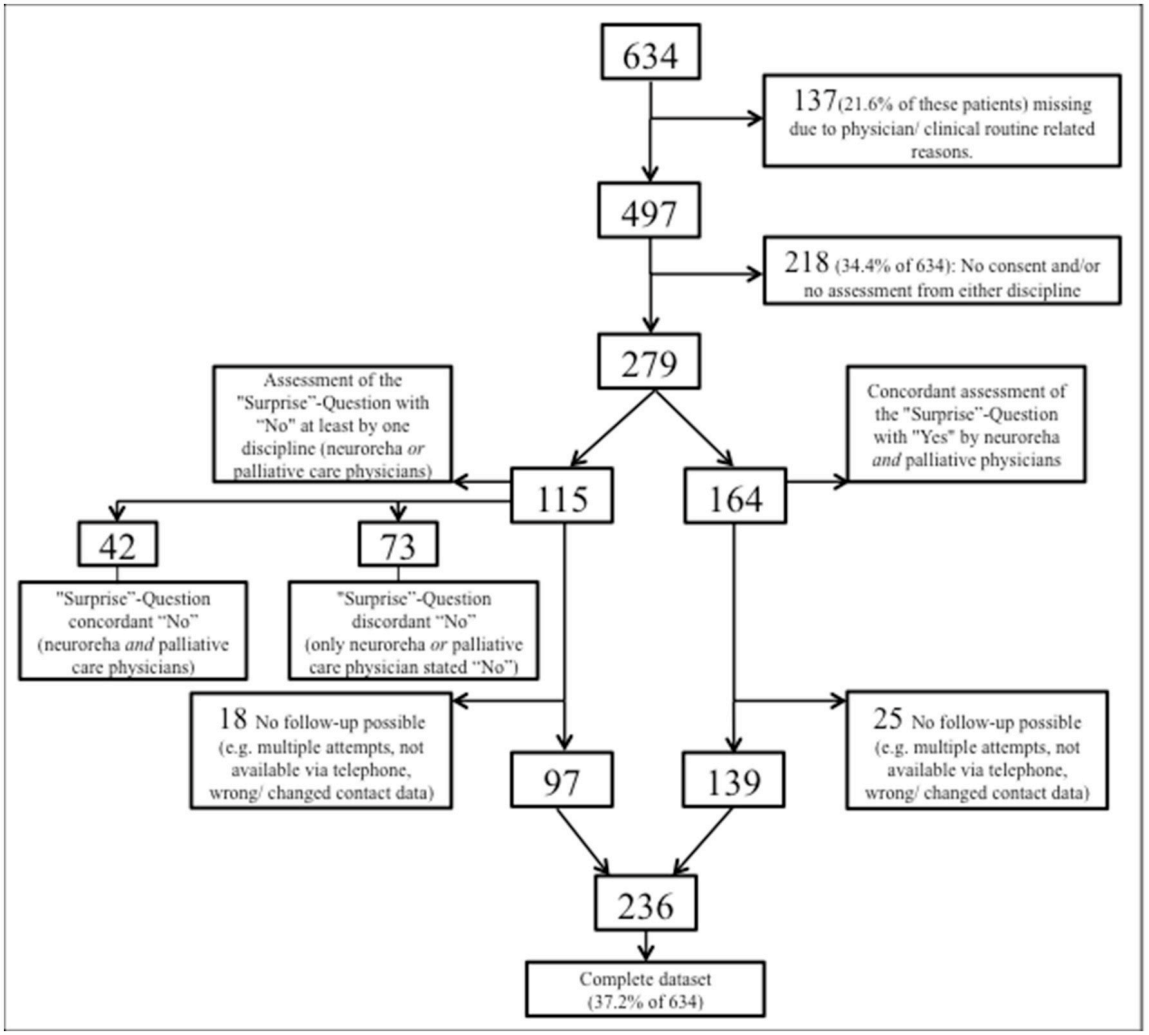
Flow-chart of study participation and follow-up.

From August 10, 2016 through March 10, 2017, 634 patients were admitted to the Dr. Becker-Rhein-Sieg-Clinic Nümbrecht, Department of neurorehabilitation (Figure 1). Of this total, 137 (21.6% of 634) could not be included into the current study due to the restricted personnel resources and tightly packed clinical routine processes. Another 218 (34.4% of 634) patients had incomplete data, i.e., either the patients (or alternatively their legal representative) did not give their informed written consent or the physicians' assessment was not attainable because of the demanding clinical routines of the physicians or patients. The remaining 279 patients were independently assessed by both NP and PCP who concordantly estimated 164 (58.8%) patients with a good prognosis (both 12-SQ “Yes”) and 42 (15.1%) patients with a poor prognosis (both 12-SQ “No”). 73 (26.2%) patients were estimated discordantly (one, either NP or PCP, answered 12-SQ “Yes”, while the other responded “No”). A total of 43 out of 279 (equaling a drop-out rate of 15.4%) patients (or alternatively their legal representative) could not be followed-up due to unattainability via the phone (Figure 1). Complete data sets were obtained from 236 patients (37.2% of 634).

### Characteristics of study participants at baseline

Demographic information of the included 279 patients can be found in Table 1 (male: 57.7%, female: 42.3%, male/female ratio: 1.4; mean age: 63 ± 14). Main diagnoses were grouped into 15 categories, secondary diagnoses were divided into seven groups (Table 1). Distribution of secondary diagnoses were as follows: 19.6% had no secondary diagnosis, 33.8% had secondary diagnoses in one category, 25.5% in two categories, 14.7% in three categories, 5.8% in four categories, and 0.7% in five categories, respectively (Table 1).

**Table 1:** Characteristics of study participants at baseline.

**Study participants**	***N*** = **279**	
Age mean (SD)	63 (14)	
Gender Male	161 (57.7%)	
Female	118 (42.3%)	
**Main diagnoses**	Ischemic stroke	131 (47.0%)
	Neurodegenerative disorders	29 (10.4%)
	Primary intracerebral hemorrhage	24 (8.6%)
	Infection of CNS	21 (7.5%)
	Multiple Sclerosis	14 (5.0%)
	Brain injury	13 (4.7%)
	Critical illness polyneuropathy	11 (3.9%)
	Spinal canal stenosis	10 (3.6%)
	Primary brain tumors	9 (3.2%)
	Subarachnoid hemorrhage	4 (1.4%)
	Slipped disc	4 (1.4%)
	Subdural hematoma	3 (1.1 %)
	Epilepsy	2 (0.7%)
	Dementia Syndrome	2 (0.7%)
	Hypoxic brain injury	2 (0.7%)
**Secondary diagnoses (categories)**	Cardiovascular diseases	184 (65.9%)
	Bronchopulmonary diseases	57 (20.4 %)
	Other internal diseases	92 (33.0) %
	Neurological and psychiatric diseases	37 (13.3 %)
	Infectious diseases	19 (6.8 %)
	Diseases of the musculoskeletal system	19 (6.8 %)
	Malignancies (except for primary brain tumors)	17 (6.1 %)
**Rehabilitation phase**	Phase B	25 (9.0%)
	Phase C	107 (38.4%)
	Phase D	147 (52.7%)

SD, standard deviation.

Comments on main diagnoses:

Primary brain tumors encompassed: Glioblastoma, Astrocytoma, Meningioma

Neurodegenerative disorders encompassed: Parkinson's disease, atypical Parkinsonian's syndromes, multiple system atrophy, amyotrophic lateral sclerosis.

Comments on secondary diagnoses:

Cardiovascular diseases encompassed: Coronary heart disease, heart failure, arterial hypertension, peripheral arterial occlusive disease, cardiac arrhythmia, heart valve diseases

Bronchopulmonary diseases encompassed: pneumonia, chronic obstructive pulmonary disease, asthma

Other internal diseases encompassed: Obesity, nutritional deficiencies, liver failure, kidney failure, diabetes mellitus and other metabolic disorders

Neurological and psychiatric diseases: Depression, psychoses, anxiety disorders, intelligence defects, e.g. post-early childhood brain damage, dementia syndrome, organic brain syndrome, multiple sclerosis, typical and atypical Parkinsonian's syndromes, polyneuropathies of various origins, epilepsy, dizziness of unknown origin, restless legs Infectious diseases encompassed: Pneumonia, urinary tract infections (including renal infections), hepatitis, thyroiditis, Lyme disease, abscesses, herpes zoster, human immunodeficiency virus

Diseases of the musculoskeletal system encompassed: fractures, osteoporosis, rheumatism, degenerative changes of the musculoskeletal system.

Rehabilitation phases B, C, D (German classification system characterizing type and intensity of neurological rehabilitation):

Phase B (early rehabilitation): There are still considerable disorders of consciousness; the ability to cooperate is severely restricted. Intensive medical treatment may be required.

Phase C (subsequent rehabilitation): High nursing need. The aim is intensive mobilization (sit up, straighten up, positioning, joint mobilization).

*Phase D (medical rehabilitation): Early rehabilitation phase is completed and possibility of actively participating in rehabilitation measurements. The aim is free walking, performing care independently and regaining everyday competence)*.

Characteristics of study participants at baseline as assessed by NP and PCP, respectively, utilizing HOPE-SP-CL, neuro supplement, and ECOG are presented in Table 2. Patients were given a higher score on all three measures when assessed by the PCP (all *p*-values < 0.025), except for feeling depressed and anxiety in the “No” group.

**Table 2:** Assessment of patients' symptom burden (HOPE-SP-CL, ECOG, neurological symptoms) by neurorehabilitation physician (NP) and palliative care physician (PCP), respectively, for the patients who were concordantly estimated to have a good prognosis (NP and PCP, *both* answered 12-SQ with “Yes”), for the patients who were concordantly assessed as having a poor prognosis (neurologist and PC physician, *both* answered 12-SQ with “No”) and for the patients whose prognosis was discordantly estimated by neurologist and palliative care physician (NP answered 12-SQ with “No” and PCP with “Yes” and vice versa, respectively).

**Symptom**	**Assessor**	**12-SQ “Yes” (NP and PCP) *N* = 164 mean (SD)**	***p*-value**	**12-SQ “No” (NP and PCP) *N* = 42 mean (SD)**	***p*-value**	**12-SQ “Yes”/ “No” (NP, PCP discordant) *N* = 73 mean (SD)**	***p*-value**	***p*- value group comparison^#^**
**HOPE-SP-CL**								
Pain	NP	0.75 (0.92)	**0.015**	0.90 (0.96)	0.743	0.55 (0.78)	0.019	0.191
	PCP	0.95 (1.17)		0.83 (1.08)		0.82 (1.18)		
Nausea	NP	0.05 (0.25)	**0.003**	0.31 (0.68)	0.414	0.15 (0.49)	0.310	0.001^*^
	PCP	0.17 (0.52)		0.45 (0.80)		0.23 (0.54)		
Vomiting	NP	0.03 (0.21)	0.046	0.26 (0.59)	0.868	0.11 (0.46)	0.885	<0.001^*^/^***^
	PCP	0.08 (0.35)		0.29 (0.60)		0.12 (0.41)		
Dyspnea	NP	0.07 (0.30)	<**0.001**	0.33 (0.65)	0.018	0.19 (0.52)	**0.003**	0.003^*^
	PCP	0.32 (0.66)		0.67 (0.85)		0.47 (0.88)		
Constipation	NP	0.25 (0.57)	**0.022**	0.64 (0.79)	0.834	0.41 (0.68)	0.114	0.002^*^/^**^
	PCP	0.38 (0.73)		0.69 (1.00)		0.60 (0.96)		
Weakness	NP	0.91 (0.77)	0.053	1.83 (0.85)	0.130	1.23 (0.95)	0.059	<0.001^*^/^**^/^***^
	PCP	1.06 (0.99)		2.05 (1.01)		1.45 (0.96)		
Loss of appetite	NP	0.24 (0.55)	0.489	0.98 (1.00)	0.878	0.68 (0.90)	0.449	<0.001^*^/^**^
	PCP	0.27 (0.63)		1.02 (1.26)		0.79 (1.09)		
Tiredness	NP	0.74 (0.69)	**0.010**	1.33 (0.90)	0.127	1.18 (0.84)	0.241	<0.001^*^/^**^
	PCP	0.98 (1.00)		1.64 (1.19)		1.32 (0.86)		
Wound care	NP	0.11 (0.44)	0.498	0.45 (0.80)	0.637	0.26 (0.67)	0.911	<0.001^*^/^**^
	PCP	0.13 (0.48)		0.48 (0.83)		0.26 (0.65)		
Assistance with activity of daily living [ADLs]	NP	0.55 (0.82)	0.041	1.76 (1.03)	0.763	1.30 (1.08)	0.878	<0.001^*^/^**^
	PCP	0.67 (0.97)		1.81 (1.27)		1.29 (1.21)		
Feeling depressed	NP	0.45 (0.70)	0.537	1.07 (1.02)	<**0.001**	0.63 (0.86)	0.104	0.045
	PCP	0.48 (0.78)		0.43 (0.77)		0.44 (0.76)		
Anxiety	NP	0.40 (0.62)	**0.009**	0.88 (0.99)	**0.002**	0.53 (0.77)	0.071	0.379
	PCP	0.55 (0.75)		0.31 (0.60)		0.36 (0.61)		
Tension	NP	0.45 (0.58)	0.144	0.83 (0.92)	0.017	0.66 (0.79)	0.054	0.416
	PCP	0.52 (0.72)		0.40 (0.70)		0.45 (0.69)		
Disorientation/ Confusion	NP	0.06 (0.29)	**0.025**	0.64 (0.79)	0.926	0.34 (0.79)	0.941	<0.001^*^/^**^/^***^
	PCP	0.13 (0.48)		0.67 (1.07)		0.34 (0.75)		
Organization of care	NP	0.13 (0.45)	<**0.001**	0.81 (0.99)	0.033	0.45 (0.83)	**0.009**	<0.001^*^/^**^
	PCP	0.34 (0.62)		1.17 (1.08)		0.88 (0.91)		
Overburdening of family	NP	0.19 (0.54)	0.129	0.64 (0.91)	0.156	0.36 (0.81)	0.014	<0.001^*^/^**^
	PCP	0.25 (0.49)		0.88 (0.97)		0.70 (0.83)		
Other symptoms	NP	0.00 (0.00)	0.317	0.07 (0.34)	1.000	0.00 (0.00)	0.180	0.031^*^
	PCP	0.01 (0.08)		0.07 (0.46)		0.05 (0.37)		
**HOPE total score**	NP	5.41 (4.02)	<**0.001**	13.74 (9.07)	0.679	9.18 (7.69)	0.035	<0.001^*^/^**^/^***^
	PCP	7.30 (5.64)		13.86 (7.14)		10.47 (6.07)		
**NEUROLOGICAL ISSUES**								
Symptoms of intracranial pressure	NP	0.09 (0.37)	0.906	0.07 (0.34)	1.000	0.04 (0.26)	1.000	0.290
	PCP	0.09 (0.37)		0.07 (0.34)		0.04 (0.26)		
Epileptic seizures	NP	0.10 (0.41)	0.039	0.14 (0.47)	0.783	0.23 (0.68)	0.161	0.790
	PCP	0.16 (0.52)		0.17 (0.66)		0.15 (0.52)		
Sensory disturbances (sensory organs)	NP	0.48 (0.77)	**0.004**	0.86 (0.98)	0.247	0.79 (0.82)	0.509	<0.001^*^/^**^
	PCP	0.65 (0.92)		1.10 (1.14)		0.85 (0.92)		
Sensation deficit (skin)	NP	0.77 (0.85)	<**0.001**	1.24 (0.98)	0.355	0.96 (0.84)	0.121	0.027^*^
	PCP	1.05 (1.02)		1.40 (1.19)		1.14 (0.99)		
Motor disturbances	NP	1.09 (0.94)	<**0.001**	1.90 (0.85)	0.168	1.41 (0.94)	0.035	<0.001^*^/^***^
	PCP	1.37 (1.02)		2.10 (1.12)		1.63 (1.15)		
Dysphagia	NP	0.07 (0.34)	**0.001**	0.74 (0.99)	0.084	0.34 (0.75)	0.435	<0.001^*^/^**^/^***^
	PCP	0.19 (0.50)		0.98 (1.22)		0.41 (0.86)		
Spasticity	NP	0.20 (0.56)	<**0.001**	0.62 (1.04)	0.061	0.37 (0.83)	0.523	0.572
	PCP	0.38 (0.78)		0.36 (0.79)		0.32 (0.69)		
Vegetative disturbances	NP	0.24 (0.59)	**0.008**	1.00 (1.01)	0.942	0.51 (0.86)	0.114	<0.001^*^/^**^/^***^
	PCP	0.37 (0.74)		1.00 (1.15)		0.71 (1.11)		
Neuropsychological disorders	NP	0.26 (0.58)	<**0.001**	0.81 (1.07)	**0.001**	0.60 (0.92)	<**0.001**	<0.001^*^/^**^
	PCP	0.66 (0.84)		1.45 (1.19)		1.26 (1.14)		
Quantitative disturbance of consciousness	NP	0.04 (0.30)	<**0.001**	0.48 (0.94)	<**0.001**	0.21 (0.62)	<**0.001**	<0.001^*^/^**^
	PCP	0.29 (0.57)		0.90 (1.01)		0.53 (0.71)		
Symptoms of delirium	NP	0.02 (0.17)	0.084	0.38 (0.73)	0.432	0.23 (0.68)	0.892	<0.001^*^/^**^
	PCP	0.06 (0.35)		0.29 (0.74)		0.21 (0.50)		
Change in personality	NP	0.09 (0.36)	<**0.001**	0.64 (0.96)	0.355	0.34 (0.73)	0.072	<0.001^*^/^**^
	PCP	0.39 (0.65)		0.79 [0.98)		0.52 (0.77)		
Loss of autonomy	NP	0.38 (0.67)	<**0.001**	1.31 (1.16)	<**0.001**	0.93 (1.10)	<**0.001**	<0.001^*^/^**^/^***^
	PCP	0.80 (0.86)		2.02 (1.07)		1.58 (1.15)		
**Neuro total score**	NP	3.89 (3.16)	<**0.001**	10.19 (7.81)	**0.001**	7.05 (6.56)	<**0.001**	<0.001^*^/^**^
	PCP	6.46 (4.05)		12.62 (8.44)		9.41 (5.88)		
**ECOG**	NP	1.28 (0.84)	<**0.001**	2.64 (1.10)	0.065	1.96 (1.02)	**0.001**	<0.001^*^/^**^/^***^
	PCP	1.52 (0.95)		2.93 (0.92)		2.33 (0.97)		
**Total score**	NP	9.30 (6.14)	<**0.001**	23.93 (16.12)	0.122	16.23 (13.51)	**0.001**	<0.001^*^/^**^/^***^
	PCP	13.76 (8.5)		26.48 (14.19)		19.88 (10.48)		

Numbers represent mean [standard deviation (SD)] and are reported here for ease of interpretation instead of median [range]. However, due to the skewed distribution of the data, non-parametric tests were applied to detect statistically significant differences. Values in bold are significant at p < 0.05 (FDR-corrected).

# If significant group differences were found (Kruskall-Wallis test), a post-hoc test was applied (FDR-corrected at p < 0.05).

* “yes” vs. “no”,

** “yes” vs. “discordant”,

*** “no” vs. “discordant”.

12-SQ, “surprise” question; PCP, palliative care physician; NP, neurorehabilitation physician; SD, standard deviation; ADLs, activity of daily living.

Significant group differences (12-SQ “Yes” by both NP *and* PCP; 12-SQ “No” by both NP *and* PCP; 12-SQ “Yes”/“No” NP and PCP discordant) were found for all but the following characteristics: pain, feeling depressed, anxiety, tension, symptoms of intracranial pressure, epileptic seizures, spasticity (all *p*-values < 0.031). As expected, *post-hoc* analyses showed that patients in the “No” group were evaluated with a higher symptom burden than patients in both the “Yes” and the “Discordant” group, and patients in the “Discordant” group were evaluated as worse than patients in the “Yes” group.

### Characteristics of deceased and surviving patients as assessed at baseline

Of the 115 patients assessed “No” on the 12-SQ by at least one discipline (Figure 1) 26 had died within the year. At the 12 months follow-up a total of 34 patients had died (also encompassing eight patients estimated as “Yes” at baseline on the 12-SQ).

Table 3 summarizes the characteristics and differences of patients still alive at the time of 12-month follow-up (*N* = 202, 85.6%) and those deceased after 12 months (*N* = 34, 14.4%). The deceased were significantly older, more often in Rehabilitation phase B, less often in Rehabilitation phase D, and suffered significantly more often from malignancies (except for primary brain tumors) (all *p*-values < 0.001). With regards to our clinical outcome measures, deceased patients were evaluated with a higher symptom burden compared to patients still alive after 12 months (all *p*-values < 0.030) (Table 3).

**Table 3:** Characteristics of patients deceased after 12 months (*N* = 34) and those still alive (*N* = 202).

	**Still alive after 12 months *N* = 202 (85.6%)**	**Deceased after 12 months *N* = 34 (14.4%)**	***p*-value**
Age mean (SD)	62 (13.7)	70.9 (13.1)	<0.001^*^
**GENDER** [**%**]			
Male	57.5	58.8	0.879
Female	42.5	41.2	0.879
**REHABILITATION PHASES** [**%**]			
B	4.0	26.5	<0.001^*^
C	37.0	52.9	0.081
D	59.0	20.6	<0.001^*^
**MAIN DIAGNOSES** [**%**]			
Ischemic stroke	48	32.4	0.256
Primary intracerebral hemorrhage	7.5	20.6	0.040
Primary brain tumors	2	11.8	0.011
Critical illness polyneuropathy	2.5	8.8	0.115
Neurodegenerative disorders	10.5	8.8	0.905
Infection of CNS	8.5	5.9	0.647
Subarachnoid hemorrhage	1.5	2.9	0.555
Subdural hematoma	1.5	/	0.561
Multiple Sclerosis	5	2.9	0.720
Epilepsy	0.5	2.9	0.245
Dementia	1	/	0.681
Hypoxic brain injury	0.5	/	0.377
Slipped disc/spinal canal stenosis	2	/	0.475
**CATEGORIES OF SECONDARY DIAGNOSES** [**%**]			
Cardiovascular diseases	64	61.8	0.240
Other internal diseases	32	35.3	0.747
Bronchopulmonary diseases	18	26.5	0.221
Malignancies (except for primary brain tumors)	4.5	23.5	<0.001^*^
Neurological and psychiatric diseases	12	8.8	0.232
Infectious diseases	5	8.8	0.092
Diseases of the musculoskeletal system	7.5	5.9	0.786
**ASSESSMENT RESULTS AT BASELINE (HOPE, NEURO SUPPLEMENT ECOG) MEAN [SD]**			
	**HOPE score t0 Survivors *N* = 202 (85.6%)**	**HOPE score t0 Deceased *N* = 34 (14.4%)**	**p-value**
**HOPE**			
Pain	0.83 (0.91]	0.74 (0.74)	0.810
Nausea	0.16 (0.39)	0.16 (0.47)	0.394
Vomiting	0.09 (0.32)	0.12 (0.35)	0.834
Dyspnea	0.23 (0.46)	0.50 (0.63)	0.004^*^
Constipation	0.37 (0.61)	0.63 (0.74)	0.030^*^
Weakness	1.11 (0.79)	1.63 (0.92)	0.001^*^
Loss of appetite	0.39 (0.64)	0.93 (0.95)	<0.001^*^
Tiredness	0.97 (0.70)	1.43 (0.75)	0.001^*^
Wound care	0.12 (0.34)	0.37 (0.63)	0.002^*^
Assistance with activity of daily living [ADLs]	0.83 (0.92)	1.66 (1.11)	<0.001^*^
Feeling depressed	0.49 (0.63)	0.66 (0.66)	0.080
Anxiety	0.48 (0.59)	0.51 (0.56)	0.564
Tension	0.50 (0.55)	0.58 (0.59)	0.427
Disorientation/Confusion	0.16 (0.42)	0.53 (0.80)	0.002^*^
Organization of care	0.37 (0.54)	0.44 (0.61)	<0.001^*^
Overburdening of family	0.31 (0.48)	0.72 (0.73)	<0.001^*^
Other symptoms	0.003 (0.04)	0.10 (0.36)	<0.001^*^
HOPE total score	7.41 (4.86)	12.15 (7.72)	<0.001^*^
**NEURO SUPPLEMENT**			
Symptoms of intracranial pressure	0.08 (0.27)	0.01 (0.09)	0.161
Epileptic seizures	0.11 (0.42)	0.21 (0.54)	0.146
Sensory disturbances (sensory organs)	0.64 (0.78)	0.87 (0.75)	0.044
Sensation deficit (skin)	0.95 (0.79)	1.41 (0.87)	0.005^*^
Motor disturbances	1.35 (0.91)	1.87 (0.96)	0.004^*^
Dysphagia	0.18 (0.42)	0.79 (1.06)	<0.001^*^
Spasticity	0.29 (0.57)	0.38 (0.71)	0.592
Vegetative disturbances	0.38 (0.67)	0.90 (1.05)	0.003^*^
Neuropsychological disorders	0.60 (0.72)	1.16 (1.01)	0.002^*^
Quantitative disturbance of consciousness	0.22 (0.39)	0.57 (0.79)	0.003^*^
Symptoms of delirium	0.07 (0.25)	0.21 (0.45)	0.015^*^
Change in personality	0.27 (0.41)	0.63 (0.76)	0.005^*^
Loss of autonomy	0.78 (0.78)	1.60 (1.05)	<0.001^*^
Neuro total score	5.97 (3.65)	10.62 (1.18)	<0.001^*^
ECOG	1.66 (0.88)	2.66 (1.09)	<0.001^*^
Total score	13.39 (7.57)	22.76 (14.15)	<0.001^*^

Numerical scores are given as mean [standard deviation]. They are reported here in lieu of medians [range] for ease of interpretation. As the data is not normally distributed, statistical group differences were analyzed with non-parametric test (FDR-corrected at p < 0.05) as directed by the data. Numerical scores for each item are presented here as mean score from both the PCP and NP.

* p < 0.05 (FDR-corrected) *SD, standard deviation; 12-SQ, “surprise” question; PCP, palliative care physician; NP, neurorehabilitation physician; ADLs, activity of daily living*.

### Prognosis estimation

*Comparison of prognosis estimation* via 12-SQ as diagnostic tool revealed an increased number of good prognoses (12-SQ “Yes”) compared to poor prognoses (12-SQ “No”) for both PCP (*p* < 0.001) and NP (*p* < 0.001). The PCP estimated more patients with a poor prognosis (12-SQ “No”) (*N* = 95) than did the NP (*N* = 62) (*p* = 0.008). Also, he offered a worse clinical assessment of patients compared to the NP. This difference is statistically significant for the total sample, the concordant “Yes” group and the discordant “Yes (NP)/No (PCP)” group (each *p* < 0.001) (Table 4).

**Table 4:** Prognosis estimation of NP and PCP.

	***N***	**Total score^+^ given by NP median [range]**	**Total score^+^ given by PCP median [range]**	***p*-value**
Total	279	10 (1–74)	15 (1–58)	<0.001^*^
12-SQ **concordant “Yes”** (NP: 12-SQ “Yes”, PCP: 12-SQ “Yes”) (concordant estimation of good prognosis)	164	8 (1–41)	13 (1–54)	<0.001^*^
12-SQ **concordant “No”** (NP: 12-SQ “No”, PCP: 12-SQ “No”) (concordant estimation of poor prognosis)	42	18.5 (2–65)	25.5 (2–58)	0.122
12-SQ **discordant** (NP: 12-SQ “No”, PCP: 12-SQ “Yes”)	20	18.5 (3–74)	22.5 (2–50)	0.737
12-SQ **discordant** (NP: 12-SQ “Yes”, PCP 12-SQ “No”)	53	11 (1–33)	19 (3–47)	<0.001^*^

+ Total score, sum of HOPE-SP-CL total score; Neuro supplement total score and ECOG.

* Indication for significant differences

*12-SQ, 12-months “surprise” question; NP, neurorehabilitation physician; PCP, palliative care physician*.

*Prognostic accuracy indices* for both disciplines are summarized in Table 5, the corresponding frequency distribution can be found in Tables 6–7. Sensitivity of the 12-SQ as stand-alone predictor was poor. While we observed a higher sensitivity for responses of the PCP relative to treating NP, this difference did not achieve statistical significance. In contrast, specificity of the 12-SQ was significantly higher when estimated by NP compared to PCP [$\chi_{\left(1,\text{N}\;=\;194\right)}^{2}=14.58$, *p* < 0.001]. There were no statistically significant differences between physicians for PPV, NPV, or AUC.

**Table 5:** Prognostic accuracy indices, 95% confidence intervals are displayed in brackets.

	**Neurorehabilitation physician**	**Palliative care physician**	**Significance**
Sensitivity	50% (0.32–0.67)	67.6% (0.50–0.83)	NS
Specificity	86.1% (0.81–0.91)	70.3% (0.64–0.77)	<0.001
PPV	37.8% (0.27–0.50)	27.7% (0.22–0.34)	NS
NPV	91.1% (0.88–0.94)	92.8% (0.89–0.96)	NS
AUC	0.68 (0.57–0.79)	0.69 (0.59–0.79)	NS
Success rate (“Yes”)	73.7%	60.2%	<0.001
Success rate (“No”)	7.2%	9.7%	NS
Success rate (combined)	80.9%	69.9%	0.002

*PPV, Positive predictive value; NPV, negative predictive value; AUC, area under the curve; NS, not significant*.

**Table 6:** Frequency table for prognosis estimation by the neurorehabilitation physician.

	**Deceased**	**Living**
12-SQ “No”	17	28
12-SQ “Yes”	17	174

**Table 7:** Frequency table for prognosis estimation by the palliative care physician.

	**Deceased**	**Living**
12-SQ “No”	23	60
12-SQ “Yes”	11	142

The combined “yes” and “no” success rate was high, with a significant difference between NP and PCP [$\chi_{\left(1,\text{N}=236\right)}^{2}$ = 9.47, *p* = 0.002] (Table 5). The success rate for the “Yes” group was also significantly higher for NP relative to PCP [$\chi_{\left(1,\text{N}=236\right)}^{2}$ = 17.80, *p* < 0.001]. Conversely, the success rate for giving a poor prognosis did not differ between physicians.

**Figure 2:**
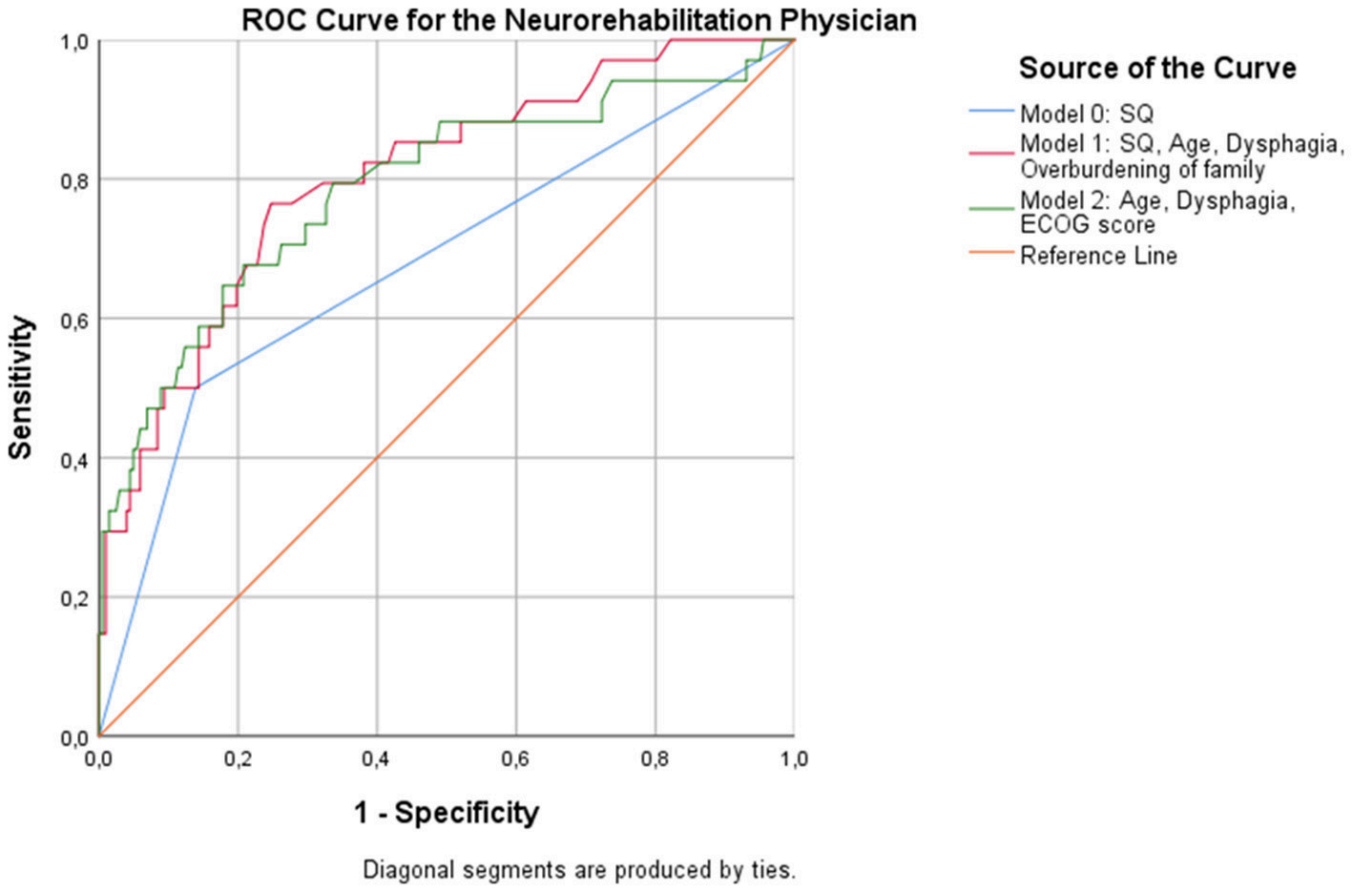
ROC curves showing the trade-off between sensitivity and specificity for all significant predictors after multivariable regression with (Model 1) and without (Model 2) the NP's response to the 12-SQ. Classification accuracy is higher for both models compared to the 12-SQ as stand-alone predictor (Model 0), though these differences did not reach statistical significance.

*Regression* analysis showed that age (*p* = 0.015), dysphagia (*p* = 0.006), and overburdening of the family (*p* = 0.036) were associated with an increased likelihood of dying at 12 months when the NP responded to the 12-SQ (Figure 2, Table 8). Overall classification was 80.3% accurate.

**Table 8:** Binary logistic regression to predict 12-month mortality as assessed by the neurorehabilitation physician.

	**Predictor**	**OR**	**AUC (one for each model)**
Model 0	12-SQ (reference: “Yes”)	6.21 (2.84–13.58)	0.68 (0.57–0.79)
Model 1	12-SQ (reference: “Yes”)	2.00 (0.75–5.33)	0.80 (0.72–0.89)
	Age	1.05 (1.01–1.09)	
	Dysphagia	2.54 (1.3–5.0)	
	Overburdening of the family	1.97 (1.05–3.7)	
Model 2	Age	1.04 (1.00–1.08)	0.79 (0.69–0.88)
	Dysphagia	2.39 (1.21–4.71)	
	ECOG score	1.90 (1.24–2.92)	

95% confidence intervals are displayed in brackets.

*OR, odds ratio; AUC, area under the curve; 12-SQ, 12-months “surprise” question; ECOG, Eastern Cooperative Oncology Group*.

**Figure 3:**
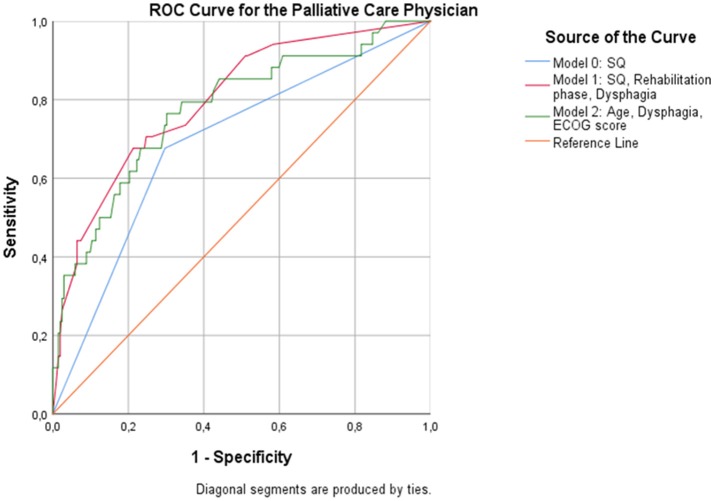
ROC curves showing the trade-off between sensitivity and specificity for all significant predictors after multivariable regression with (Model 1) and without (Model 2) the PCP's response to the 12-SQ. Classification accuracy is higher for both models compared to the 12-SQ as stand-alone predictor (Model 0), though these differences did not achieve statistical significance.

When patients were assessed by the PCP, the overall predictive accuracy of the model was 79.9%. Response to the 12-SQ (*p* = 0.014), dysphagia (*p* = 0.041), and rehabilitation phase (*p* = 0.014) were statistically associated with 12-month mortality. Patients in the “No” group were 3 times more likely to die than patients in the “Yes” group. Rehabilitation phase also predicted the likelihood of dying at 12 months with patients in phase B registering as 7.3 times more likely to die than patients in phase D (*p* = 0.005), and patients in phase C being 2.8 times more likely to die compared to patients in phase D (*p* = 0.041) (Figure 3, Table 9).

**Table 9:** Binary logistic regression to predict 12-month mortality as assessed by the Palliative Care Physician.

	**Predictor**	**OR**	**AUC (one for each model)**
Model 0	12-SQ (reference: “Yes”)	4.95 (2.27–10.79)	0.69 (0.59–0.79)
Model 1	12-SQ (reference: “Yes”)	2.95 (1.25–6.97)	0.80 (0.72–0.88)
	Rehabilitation phase (reference: phase D)		
	Rehabilitation phase B	7.32 (1.83–29.26)	
	Rehabilitation phase C	2.78 (1.04–7.39)	
	Dysphagia	1.61 (1.02–2.54)	
Model 2	Age	1.04 (1.00–1.07)	0.78 (0.69–0.87)
	Dysphagia	1.61 (1.05–2.47)	
	ECOG score	1.87 (1.21–2.88)	

95% confidence intervals are displayed in brackets.

*OR, odds ratio; AUC, area under the curve; 12-SQ, 12-months “surprise” question; ECOG, Eastern Cooperative Oncology Group*.

Without the 12-SQ as relevant predictor, age (NP: *p* = 0.038; PCP: *p* = 0.026) and dysphagia (NP: *p* = 0.012, PCP: *p* = 0.029) remained significant predictors, irrespective of the physicians' medical background. In addition, for both the NP and the PCP, an increased ECOG score was significantly related to an increased risk of dying (NP: *p* = 0.003; PCP: *p* = 0.005) (Figures 2–3, Tables 8, 9). When assessed by the PCP, the model showed 86.9% overall classification accuracy, which increased to 89% when assessed by the NP.

## Discussion

According to literature search this is the first study investigating prognosis estimation using 12-SQ and assessment of palliative care symptoms supplemented by neurological items, as rated by NP and PCP, respectively, in a sample of neurorehabilitation patients.

Prognosis estimation in this patient group proved challenging when utilizing 12-SQ as a single tool, which was reflected in poor prognostic accuracy indices, found also for other non-cancer diseases (21, 23). However, in our study, answering 12-SQ “No” pointed to physicians' expectation of poor prognosis as both treating NP as well as the PCP evaluated the 12-SQ “No” group consistently with the highest symptom burden. Overall, treating NP assessed patients better (meaning lower scores on the utilized measures) than the PCP. A potential explanation might be the clinical background of assessors with PCP primarily caring for the potential of general deterioration and the end of life and the NP being more concerned with recovery and restitution. Seemingly combined expertise might be needed for a balanced and accurate estimation.

In our study, accurate prediction for patients at increased risk of dying was especially low for NP. Accordingly, the NP demonstrated higher accuracy for predicting whether patients would still be alive after 12 months compared to PCP. Our results suggest the use of “12-SQ2”: “Would I be surprised if this patient is still alive after twelve months?” (45) for physicians with a background in neurology or a combination of the original 12-SQ and the 12-SQ2, which has been piloted in a sample of general practitioners (45, 46).

Significantly, the 12-SQ was not originally developed for an accurate prognosis in the prediction of death, but to identify patients in need of palliative care (1–19). In specialties such as neurorehabilitation the implementation of the 12-SQ in combination with a palliative care assessment into the clinical routine—as in our study—might help sensitize healthcare professionals toward palliative care issues like initiating conversation on advanced care planning or prognosis or integrating additional services like palliative and hospice care services if needed. Currently, this approach is not yet well recognized in German neurorehabilitation and integrative prognostic studies may serve to help change this, an eventual consequence which would be beneficial to both patients and the caregivers involved in neurorehabilitation. The need for such a multi-disciplinary neuropalliative rehabilitation approach has already been highlighted and recommended in the UK's National Service Framework for Long-term (Neurological) Conditions (29–31) but has not been consistently pursued in neurorehabilitation in Germany so far.

In a recent study, the 12-SQ was combined with further clinical parameters to better identify patients with palliative care needs and aid in prognosis estimation (22). Our study corroborates the importance of bringing in additional clinical assessments to the 12-SQ, i.e., HOPE-SP-CL, neuro supplement, ECOG, diagnoses, age, gender, rehabilitation phase, to establish a broader basis for estimation of prognosis and palliative care needs.

With the help of the additional data we were able to identify several items (HOPE-SP-CL as well as neuro supplement as well as ECOG) which were scored significantly higher at baseline (meaning worse) for the group of patients who died after 12 months compared to those still alive. This speaks in favor of these measurements being suitable to assess patients' deteriorating general health condition. Moreover, the regression identified three factors (age, ECOG, dysphagia) which might help to predict one-year mortality in our sample of neurorehabilitation patients. These three factors are all reasonable indicators for a worsened overall condition. As anticipated, increased age is a risk factor for dying, even more so when seriously ill. Second, an increasing ECOG score in patients indicates decreasing, i.e., worse, functionality in all daily activities. Lastly, dysphagia has been identified as a critical prognostic factor in neurological patients, especially those suffering from stroke and neurodegenerative disorders (20, 47, 48). In the rehabilitation setting mortality risk increased by a factor of 13 for patients suffering from dysphagia (47). Depending on subtypes, patients suffering from progressive supranuclear palsy or multiple system atrophy died 2–24 months after developing severe dysphagia (48). Potential reasons for dysphagia being associated with a poor prognosis might be the development of serious complications like aspiration pneumonia (20, 47, 48).

As the 12-SQ is a commonly used tool for estimation of prognosis—even if poor when used as the only instrument—and for initiating palliative care in cancer and non-cancer patients (1–19), we investigated whether adding further clinical characteristics to the 12-SQ would improve the overall predictive power. Again, our results indicate that age and dysphagia, as well as rehabilitation phase and overburdening of the family in combination with the 12-SQ have great prognostic value in estimating prognosis and thus identifying patients in need of palliative care. These two additional factors can be interpreted similarly to the ECOG: Patients in rehabilitation phases C and B suffer from a more serious illness with decreased functionality compared to phase D and “overburdening of family” also indicates patients' health deterioration. It is well known that as patients' health condition worsens, family caregivers physically and psychologically reach their limits (49–52).

## Limitations

Of our initial sample of 634 patients only 236 (37%) could be included and later followed up. This proportion is quite good for a palliative care study, but generalizability remains limited as we were unable to present a full data set. Moreover, study participants attending rehabilitation phase B (i.e., seriously ill patients) were represented to a lesser degree than patients in rehabilitation phase C or D. One potential reason might be the increased difficulty in obtaining consent (seriously ill, legal representative, etc.). Of the 236 included and followed-up patients 14% died within one year. Despite similar incidences of death reported in other studies investigating the 12-SQ (21) this is a moderate to small fraction complicating the interpretation of prognostic accuracy indices. The neurorehabilitation population investigated was quite heterogeneous. Group sizes of the different main diagnoses groups were unequal ranging from 47% (ischemic stroke, largest group) to 0.2% (epilepsy, dementia syndrome, and hypoxic brain injury, respectively) and thus, a sound subgroup analysis was not possible. At least from results of this study, we cannot conclude whether the 12-SQ and identified risk factors may be of differing predictive accuracy with respect to special disease entities s (e.g., ALS representing a progressive disorder vs. ischemic stroke normally representing a monophasic illness). One caveat to the interpretation of our results is that various NPs (each time the respective treating NP) evaluated the patients while there was a single, external, consulting PCP assessing the patients, so that systematic assessment bias for the PCP could not be averaged out. That clinical background influenced 12-SQ estimation was also apparent amongst the rather large group of patients who were discordantly judged using the 12-SQ (26.2%) thereby reducing the number of unambiguously assigned patients.

## Conclusion

Prognosis estimation of neurological patients is challenging and thus, identifying the right point in time to integrate the palliative care approach for neurological patients remains difficult. Implementing an assessment tool into the care of these patients - in the current study with a sample of neurorehabilitation patients - combining the 12-SQ with palliative care and neurological items might improve predictive performance of 12 months survival and thus identify an appropriate, sufficient time to initiate the palliative care approach and services if needed. Factors improving predictive accuracy (with and without the 12-SQ) were rehabilitation phase, dysphagia, age, overburdening of family and ECOG. Professional background influences assessment and prognosis estimation.

## Author contributions

ME, HG, RV, AK, and IB planned the study. ME and AK recruited and assessed patients. KD, AK, IB, and HG analyzed the data. HG, KD, AK, ME, IB, and RV wrote and corrected the manuscript.

### Conflict of interest statement

The authors declare that the research was conducted in the absence of any commercial or financial relationships that could be construed as a potential conflict of interest. The handling editor is currently organizing a Research Topic with one of the authors RV, and confirms the absence of any other collaboration.

## Footnotes

^1^http://www.awmf.org/uploads/tx_szleitlinien/128-001OLl_S3_Palliativmedizin_2015-07.pdf (accessed 29.05.2018)

^2^https://www.hope-clara.de/download_1/ (accessed 29.05.2018)

^3^http://www.who.int/cancer/palliative/definition/en/ (accessed 29.05.2018)

## Abbreviations

12-SQ: twelve-months “surprise”question
PC: palliative care
NP: neurorehabilitation physicians
PCP: palliative care physician
HOPE = HOspiz- und PalliativErhebung: German Hospice and Palliative Care Evaluation initiative
HOPE-SP-CL: HOPE symptom and problem checklist
ECOG: Eastern Cooperative Oncology Group
POS: palliative outcome scale
COPD: chronic obstructive pulmonary disease
LTNC: long-term neurological conditions
WHO: World Health Organization
ROC: receiver operating characteristics
PPV: positive predictive value
NPV: negative predictive value
AUC: area under the curve
FDR: false discovery rate
SPSS: Statistical Package for the Social Sciences
SD: standard deviation
ADLs: activity of daily living
OR: odds ratio.
